# Metformin Protects ARPE-19 Cells from Glyoxal-Induced Oxidative Stress

**DOI:** 10.1155/2020/1740943

**Published:** 2020-07-09

**Authors:** Sichang Qu, Chaoyang Zhang, Dandan Liu, Jing Wu, Haibin Tian, Lixia Lu, Guo-Tong Xu, Fang Liu, Jingfa Zhang

**Affiliations:** ^1^Department of Ophthalmology of Shanghai Tenth People's Hospital, Tongji Eye Institute, Department of Regenerative Medicine, And Department of Pharmacology, Tongji University School of Medicine, Shanghai, China; ^2^Department of Ophthalmology, Shanghai General Hospital (Shanghai First People's Hospital), Shanghai Jiao Tong University, Shanghai, China; ^3^National Clinical Research Center for Eye Diseases, Shanghai, China; ^4^Shanghai Key Laboratory of Ocular Fundus Diseases, Shanghai, China; ^5^Shanghai Engineering Center for Visual Science and Photomedicine, Shanghai, China; ^6^Shanghai Engineering Center for Precise Diagnosis and Treatment of Eye Diseases, Shanghai, China

## Abstract

The protective effects and mechanisms of metformin against oxidative stress were evaluated both *in vivo* and *in vitro*. ARPE-19 cells comprised the normal group, the glyoxal-treated group (0.5 mM glyoxal), and the glyoxal+metformin group (0.5 mM glyoxal and 0.1 mM metformin). In the *in vitro* model, differences in cell viability, ROS production, NO products, cellular apoptosis, and the expressions of phospho-AMPK*α*, total-AMPK*α*, Sirt1, Nrf2, TXNIP, ZO-1, and Occludin were assessed. In the glyoxal-treated group, cell viability and NO production were decreased, while ROS production and cell apoptosis were increased (*p* < 0.05), compared with the control group. These changes were prevented by metformin treatment. Protein expressions of phospho-AMPK*α*, Sirt1, TXNIP, ZO-1, and Occludin, but not Nrf2, were decreased significantly in the glyoxal-treated group compared to normal controls. Metformin treatment significantly increased the above protein expressions and slightly increased TXNIP expression. Immunofluorescence showed that metformin prevented the glyoxal-induced, disorganized tight junctions in ARPE-19 cells. To confirm metformin's protection, Sprague-Dawley rats were injected intravenously with sodium iodate (SI) to induce oxidative stress in the retinal pigment epithelium (RPE). Metformin was then delivered intraperitoneally or intravitreally. One day and three days after SI and metformin treatments, the RPE-Bruch's membrane-choriocapillaris complex was isolated and immune-stained with ZO-1 antibodies. The morphology of the RPE showed enlarged cellular bodies and disorganized ZO-1 staining in SI-treated rats. Metformin treatment prevented these changes. The results indicated that metformin maintained the barrier functions of RPE cells both *in vivo* and *in vitro*. Metformin exerted its protection against oxidative stress possibly via activating AMPK/Sirt1 and increasing TXNIP. Metformin has been proposed as a candidate drug for age-related macular degeneration (AMD) by both preclinical and clinical studies. The cellular and animal models used in this study might be useful for the interpretation of the molecular mechanisms involved in the drug activity.

## 1. Introduction

Age-related macular degeneration (AMD) is the leading cause of severe vision loss and blindness in elderly people around the world. According to epidemiological surveys, the global prevalence of any type of AMD is around 8.7% [1], and the prevalence is even higher among Europeans over the age of 70 years (at 16.2%), [2] which is estimated to increase by 15% by 2050 [3]. Multiple environmental factors and genetics are associated with AMD. The pathological mechanisms of AMD are complex, including immune dysfunction, complement and inflammation activation, oxidative stress, mitochondrial damage, and abnormal lipid metabolism [4].

Of these mechanisms, oxidative stress seems to be an important contributing factor to the development of AMD, especially in its dry form. With a two-level model hypothesis for AMD, oxidative stress is considered as the first level of molecular damage, which can then cause the second-level oxidative burst and inflammation [5]. During disease progression, aging contributes most to reactive oxygen species (ROS) accumulation, leading to sustained damage to the retina. Regarding epigenetics, DNA methylation of glutathione S-transferase isoforms mu1 (GSTM1) and mu5 (GSTM5) has been reported to increase the susceptibility to oxidative stress in patients with AMD [6, 7], and cigarette smoke treatment of retinal pigment epithelium (RPE) decreased chromatin accessibility, also providing an epigenetic link between a known risk factor for AMD and AMD pathology [8]. According to our previous proteomics analysis [9], aqueous humor proteins with antioxidant properties, including protein S100-A8, carbonic anhydrase, and L-lactate dehydrogenase A chain, were found to be upregulated in patients with dry AMD, indicating oxidative stress played a role in the pathogenesis of AMD.

As the first-line drug treatment for type 2 diabetes, metformin has been widely used for decades and has recently become a “miracle” drug for its extensive usefulness. It is a synthetic derivative of a natural product, galegine, and not designed for any specific pathway [10]; therefore, it has the potential to treat multiple diseases. Studies have shown that metformin has great beneficial effects in cardiovascular diseases, cancer, immune inflammatory diseases, and aging. [11–13] It has a variety of biological roles in treating these diseases, including regulating protein metabolism, repairing DNA damage, and transporting and repositioning metal ions [14]. In addition, retrospective studies have shown that metformin can significantly lower the risk of AMD or slow down progression. [15, 16] However, the underlying mechanisms of metformin have not been fully understood.

This study explored whether metformin could protect the RPE from oxidative stress both *in vivo* and *in vitro*, mainly focusing on glyoxal-treated ARPE-19 cells and the possible mechanism(s) of the effects. We thus provided support for its potential use as a future treatment option for dry AMD.

## 2. Materials and Methods

### 2.1. Experimental Animal Modeling

Male Sprague-Dawley (SD) rats (about 200 g body weight) were purchased from Slaccas (Shanghai, China) and raised under 12 hours of light/12 hours of dark cycles. The study followed the Association for Research in Vision and Ophthalmology (ARVO) Statement for the Use of Animals in Ophthalmic and Vision Research.

SD rats were injected intravenously with sodium iodate (SI) solution (Sigma, USA, dissolved in normal saline, 40 mg/kg body weight) to induce RPE oxidative stress in the rat model. To test the protective function of metformin in SI-treated rats, the rats were injected simultaneously with metformin, which was delivered either intraperitoneally (metformin dose 200 mg/kg body weight) or intravitreally (5 *μ*g metformin dissolved in 2 *μ*L normal saline). Normal rats received an equivalent volume of normal saline and served as controls. One day or 3 days after SI injection, the rats were killed, and the eyes were enucleated. The eyes were fixed in 1x Phosphate Buffer Saline- (PBS-) buffered 4% paraformaldehyde solution and stored at 4°C before use. The RPE-Bruch's membrane-choriocapillaris complexes (RBCCs) from different groups were isolated carefully under a dissection microscope, and then the immunofluorescence of ZO-1 was measured according to our previous study [17].

### 2.2. Cell Culture

ARPE-19 cells were cultured in Dulbecco's Modified Eagle Medium/Factor 12 (DMEM/F12) medium (Gibco) with 10% fetal bovine serum (Gibco) and 1% penicillin/streptomycin (Invitrogen) at 37°C with 5% CO_2_ in a humidified incubator. The cells were divided into three groups: normal control (N), glyoxal (0.5 mM)-treated group (G), and glyoxal (0.5 mM)+metformin (0.1 mM)-treated group (G+M).

### 2.3. Cell Viability Assay

Cell viability was assayed with a Cell Counting Kit-8 (CCK8, YEASEN 40203ES60) according to the manufacturer's instructions. ARPE-19 cells were seeded on 96-well plates at a density of 1,000 cells per well and treated with glyoxal and metformin for the indicated times. For the cell viability assay, the culture medium was replaced with new medium containing 10 *μ*L CCK8 solution at 37°C for 3 h. The optical density (OD) was measured with a microplate spectrophotometer (Tecan, Crailsheim, Germany) at an absorbance wavelength of 450 nm. The OD was normalized according to the normal controls.

### 2.4. Detection of Intracellular ROS

Intracellular ROS formation was detected using 2′,7′-dichlorodihydrofluorescein diacetate (DCFH-DA) probes according to the manufacturer's instructions (S0033, Reactive Oxygen Species Assay Kit, Beyotime, China). ARPE-19 cells were seeded on 96-well plates (for quantitation measurement) or on cell slides (for fluorescence detection). The cells were incubated with 10 *μ*M DCFH-DA at 37°C for 30 min; after washing three times, the fluorescence intensity was measured with a multifunctional microplate reader (BioTek Synergy4, USA) with an excitation wavelength of 485 nm and emission wavelength of 528 nm. The cells incubated with Rosup served as positive controls. For immunofluorescence measurement, the intracellular ROS in cells on slides were visualized with a Leica microscope (DMI3000, Germany).

### 2.5. Determination of NO Production

Nitric oxide (NO) levels were measured according to the Griess method by detecting NO_3_^−^ and NO_2_^−^ with a NO Kit (S0021, Beyotime, China). Standard samples were diluted into different concentrations (0, 1, 2, 5, 10, 20, 40, 60, and 100 *μ*M) to plot the standard curve. After the above treatment, 50 *μ*L of the culture supernatant fluid was collected and 50 *μ*L (per well) Griess Reagent I and 50 *μ*L (per well) Griess Reagent II were added. Then, NO production was measured using OD value readings with a microplate spectrophotometer (Tecan, Crailsheim, Germany) at an absorbance wavelength of 540 nm. The concentrations were calculated from the standard curve.

### 2.6. TUNEL Assay

Cell death was detected with a terminal deoxynucleotidyl transferase-mediated dUTP nick-end labeling (TUNEL) assay (*In Situ* Cell Death Detection Kit, Roche, Switzerland) according to the manufacturer's instructions. The air-dried cell slides were fixed with 4% paraformaldehyde for 30 min at room temperature, and permeabilized with 0.5% Triton X-100 for 10 min. After three rinses with PBS, 100 *μ*L of the TUNEL reaction mixture was added, and the slides were incubated in a humidified atmosphere for 1 h at 37°C in the dark. For the negative controls, only the Label solution was added, and for the positive controls, the slides were pretreated with DNase I for 10 min at room temperature. The slides were analyzed under a fluorescence microscope (DMI3000, Germany) with an excitation wavelength of 488 nm.

### 2.7. Protein Extraction and Western Blot

ARPE-19 cells were lysed with RIPA buffer containing phenylmethanesulfonyl fluoride (PMSF) and phosphatase inhibitors on ice for 30 min. The concentration was measured with a Pierce bicinchoninic acid (BCA) Protein Assay Kit (Thermo Scientific). Equal amounts of protein were resolved on 10% or 15% SDS-polyacrylamide gels and transferred onto nitrocellulose membranes (Bio-Rad, Shanghai, China). The membranes were blocked with 5% bovine serum albumin (BSA) diluted in 1×Tris buffered saline with Tween-20 (TBST) at room temperature for 30 min and then incubated with the primary antibodies against phospho-AMPK*α* (Cell Signaling Technology, 2535, 1 : 1,000), Nrf2 (Cell Signaling Technology, 12721, 1 : 1,000), Sirt1 (Cell Signaling Technology, 9475, 1 : 1,000), TXNIP (Invitrogen, 40-3700, 1 : 1,000), ZO-1 (Invitrogen, 61-7300, 1 : 500), Occludin (Invitrogen, 33-1500, 1 : 500), or *β*-actin (Cell Signaling Technology, 3700, 1 : 5,000), separately, at 4°C overnight with gentle shaking. After washing with TBST three times, the membranes were incubated with the corresponding secondary antibodies (LICOR Biosciences, 925-68023/925-32210) at room temperature for 1 h. After extensive washing with TBST, the membranes were imaged with an Odyssey infrared imaging system (LICOR Biosciences, Lincoln, NE, USA). For AMP-activated protein kinase (AMPK), after phospho-AMPK*α* detection, the membrane was stripped with the stripping buffer and then the primary antibody against total-AMPK*α* (Cell Signaling Technology, 5832, 1 : 1,000) was added and incubated. The OD of each band was quantified and normalized with *β*-actin.

### 2.8. Immunofluorescence

The immunofluorescence measurements were performed as previously described [18]. RBCCs and ARPE-19 cells were permeabilized with 0.5% Triton X-100 for 10 min, blocked with TBST containing 5% BSA for 1 h (for RBCCs) and 30 min (for ARPE-19 cells) at room temperature, and incubated with anit-ZO-1 antibodies (Invitrogen, 61-7300, 1 : 100 for RBCCs and 1 : 200 for ARPE-19 cells) at 4°C overnight. After washing thoroughly with PBS, RBCCs and ARPE-19 cells were incubated with the corresponding secondary antibodies (Abcam, ab150065) at room temperature for 1 h and counterstained with 4,6-diamidino-2-phenylindole (DAPI, YEASEN, 40727ES10) for 3 min. The RBCCs and slides were observed under a confocal microscope (Zeiss, Germany) with an excitation wavelength of 488 nm and emission wavelength of 495-532 nm.

### 2.9. Statistics Analysis

The data were analyzed with a one-way ANOVA followed by Student's test, and were expressed as the mean ± SE (standard error). A *p* value less than 0.05 was considered statistically significant.

## 3. Results

### 3.1. Metformin Protected ARPE-19 Cells against Glyoxal-Induced Cytotoxicity

**Figure 1:**
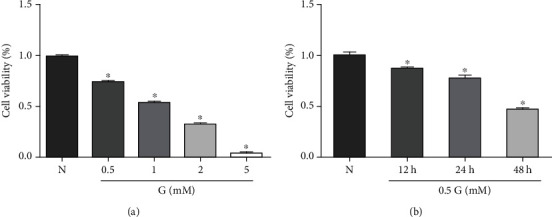
Cell viability assay for ARPE-19 cells treated with glyoxal. (a) ARPE-19 cells were treated with varying concentrations of glyoxal (0.5, 1, 2, and 5 mM) for 24 h. (b) ARPE-19 cells were treated with 0.5 mM glyoxal for 12, 24, and 48 h. Cell viability was measured with CCK8. Data are expressed as the mean ± SE, *n* = 8 in (a), *n* = 5 in (b). ∗*p* < 0.05 compared with the normal group. N: normal control; G: glyoxal-treated group.

Oxidative stress plays an important role in the pathogenesis of AMD. To study the effects and possible mechanisms of metformin, ARPE-19 cells treated with glyoxal were employed as an *in vitro* model to mimic oxidative stress in dry AMD. To establish the optimal conditions for glyoxal-treated-ARPE-19 cells, ARPE-19 cells were treated with different concentrations of glyoxal (0.5, 1, 2, and 5 mM) for 24 h. As shown in Figure 1(a), cell viability was significantly decreased in a dose-dependent manner with increased concentrations of glyoxal. Cell viability was decreased by 24.90% (0.5 mM), 46.23% (1 mM), 66.76% (2 mM), and 95.26% (5 mM), respectively. Based on these results, glyoxal with 0.5 mM was selected. With this concentration of glyoxal, ARPE-19 cells were incubated for 12, 24, and 48 h, and then cell viability was assayed. The data showed that cell viability was decreased by 12.34% (12 h), 23.24% (24 h), and 53.61% (48 h), respectively (Figure 1(b)). ARPE-19 cells treated with 0.5 mM glyoxal for 24 h were then used in the following experiments.

**Figure 2:**
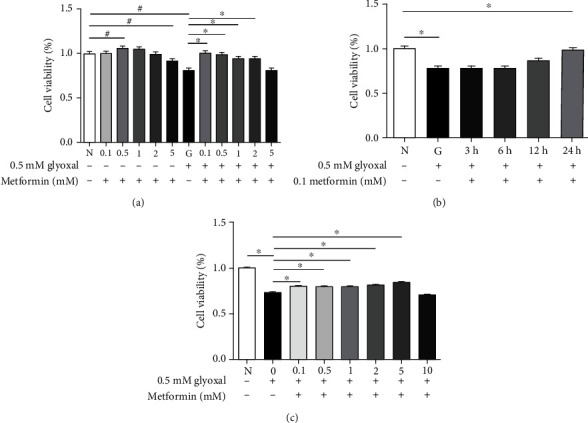
Cell viability assay for ARPE-19 cells treated with glyoxal and metformin. (a) ARPE-19 cells were incubated in varying concentrations of metformin (0.1, 0.5, 1, 2, and 5 mM) for 24 h or incubated in varying concentrations of metformin (0.1, 0.5, 1, 2, and 5 mM) and 0.5 mM glyoxal for 24 h. (b) ARPE-19 cells were incubated with 0.5 mM glyoxal for 24 h and 0.1 mM metformin was added in the last 3, 6, or 12 h or was added simultaneously. (c) ARPE-19 cells were pretreated with varying concentrations of metformin (0, 0.1, 0.5, 1, 2, 5, and 10 mM) for 12 h and then incubated with the indicated doses of metformin and 0.5 mM glyoxal for another 24 h. Cell viability was measured with CCK8. Data are expressed as the mean ± SE (*n* = 10 in (a), *n* = 5 in (b), and *n* = 7 in (c)). ∗*p* < 0.05 versus glyoxal group, ^#^*p* < 0.05 versus glyoxal group. N: normal control; G: glyoxal-treated group.

To find the optimal dosage and treatment time of metformin, ARPE-19 cells with or without glyoxal incubation were treated with different concentrations of metformin (0.1, 0.5, 1, 2, and 5 mM) for 24 h. As shown in Figure 2(a), a low concentration of metformin had little impact on cell viability: 0.26% (*p* > 0.05, 0.1 mM) and 2.10% (*p* > 0.05, 2 mM) decrease or 5.37% (*p* < 0.05, 0.5 mM) and 3.98% (*p* > 0.05, 1 mM) increase. In contrast, a high concentration of metformin could decrease cell viability by 8.75% (*p* < 0.05, 5 mM). However, under glyoxal treatment, metformin with concentrations from 0.1 to 2 mM could increase cell viability significantly: i.e., by 24.13% (*p* < 0.05, 0.1 mM), 21.06% (*p* < 0.05, 0.5 mM), 16.54% (*p* < 0.05, 1 mM), 15.66% (*p* < 0.05, 2 mM), and 0.15% (*p* > 0.05, 5 mM; Figure 2(a)). Combining the effect of metformin in both normal controls and glyoxal-treated ARPE-19 cells, the cells were treated with 0.1 mM metformin for different time periods before the glyoxal treatment and were harvested after 24 h. As shown in Figure 2(b), cell viability was increased with extended treatment times of metformin: i.e., by 1.53% (*p* > 0.05, 3 h), 1.05% (*p* > 0.05, 6 h), 10.19% (*p* > 0.05, 12 h), and 27.60% (*p* < 0.05, 24 h).

We also pretreated ARPE-19 cells with varying concentrations of metformin (0, 0.1, 0.5, 1, 2, 5, and 10 mM) for 12 h, and then we incubated the cells with 0.5 mM glyoxal for another 24 h to determine whether metformin could still protect ARPE-19 cells against glyoxal insult. The results showed that cell viability increased by 9.71% (*p* < 0.05, 0.1 mM), 8.89% (*p* < 0.05, 0.5 mM), 9.54% (*p* < 0.05, 1 mM), 11.80% (*p* < 0.05, 2 mM), 15.27% (*p* < 0.05, 5 mM), and decreased by 4.07% (*p* < 0.05, 10 mM) (Figure 2(c)).

Combining the above results, we determined that glyoxal (0.5 mM) treatment of ARPE-19 cells and 0.5 mM glyoxal coculturing with 0.1 mM metformin for 24 h were optimal conditions in this *in vitro* system.

### 3.2. Metformin Reduced Apoptosis in Glyoxal-Treated ARPE-19 Cells

**Figure 3:**
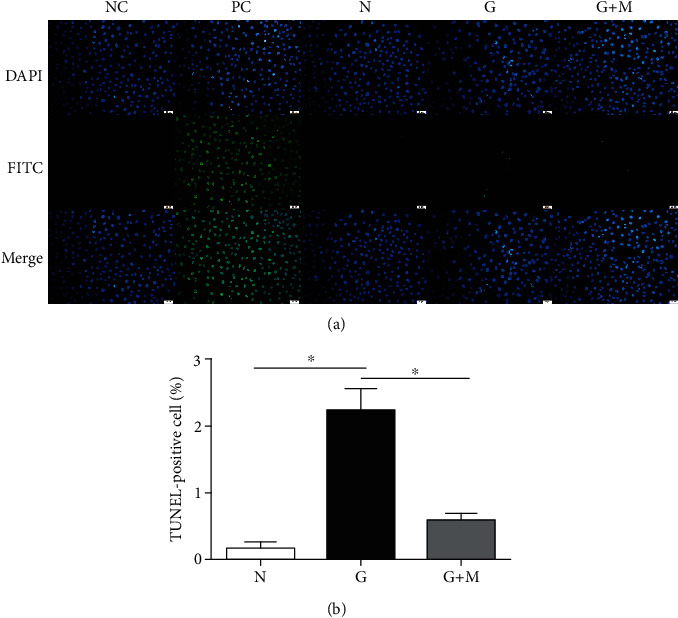
Metformin reduced apoptosis of ARPE-19 cells induced by glyoxal. Glyoxal-treated ARPE-19 cells were treated with or without metformin (0.1 mM) for 24 h. The apoptotic cells were detected with a TUNEL assay. TUNEL-positive cells were stained in green and nuclei were counterstained in blue. The slides were observed under a fluorescent microscope (a) and quantified (b). Data are expressed as the mean ± SE (*n* = 5, ∗*p* < 0.05 versus glyoxal group). NC: negative control; PC: positive control; N: normal control; G: glyoxal-treated group; and G+M: glyoxal and metformin cotreated group. Scale bar, 20 *μ*m.

We performed a TUNEL assay to determine whether metformin increased cell viability because of its effect against cell apoptosis. As shown in Figure 3, the number of apoptotic cells was significantly increased in the glyoxal-treated group. Cell apoptosis was largely prevented by metformin. Compared with normal controls (0.17%), the ratio of TUNEL-positive cells was increased significantly (2.22%, *p* < 0.05) and was decreased significantly by metformin (0.57%, *p* < 0.05, Figure 3(b)).

### 3.3. Metformin Decreased ROS Production and Increased NO Levels

**Figure 4:**
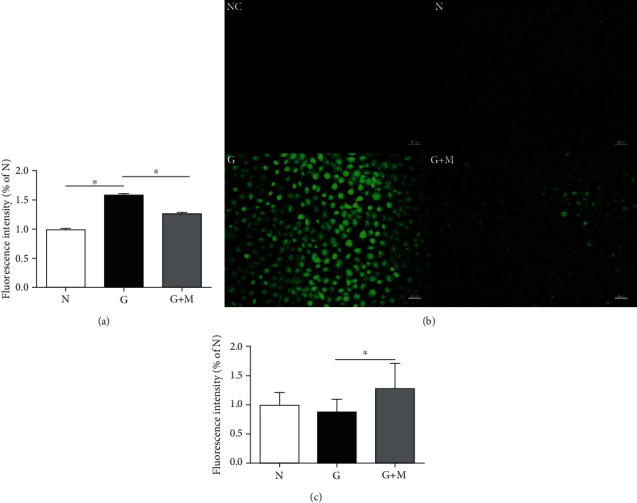
Metformin treatment reduced ROS production and increased the NO levels in glyoxal-treated ARPE-19 cells. Glyoxal-treated ARPE-19 cells were treated with or without metformin (0.1 mM) for 24 h. The ROS production was quantified with a Reactive Oxygen Species Assay Kit (a) and immunofluorescence (b). The NO products were quantified with a Nitric Oxide Kit (c). Data are expressed as the mean ± SE (*n* = 10 in (a), *n* = 8 in (c)), ∗*p* < 0.05 versus glyoxal group). NC: negative control, cells treated with DMEM/F12 without a probe. N: normal control; G, glyoxal-treated group; G+M, glyoxal and metformin cotreated group. Scale bar, 20 *μ*m.

Since glyoxal is a reactive intermediate for advanced glycation end products (AGEs), ROS can form, and this can ultimately lead to intracellular oxidative stress. ROS production has been detected in glyoxal-treated ARPE-19 cells with or without metformin. Study results showed that glyoxal treatment significantly increased the production of intracellular ROS, about 1.6-fold of normal controls, and metformin significantly attenuated this increase (Figure 4(a)). The differential intensity of green fluorescence (Figure 4(b)) in the three groups also confirmed the result. In addition, NO levels were decreased in the glyoxal-treated group compared with normal controls, which had been increased by metformin. The NO levels were decreased by 11.46% (*n* = 8, *p* > 0.05) in the glyoxal-treated group and increased by 45.49% (*n* = 8, *p* < 0.05) after metformin treatment (Figure 4(c)).

### 3.4. Metformin Activated the AMPK/Sirt1 Signaling Pathway and Increased Nrf2 and TXNIP in Glyoxal-Treated ARPE-19 Cells

**Figure 5:**
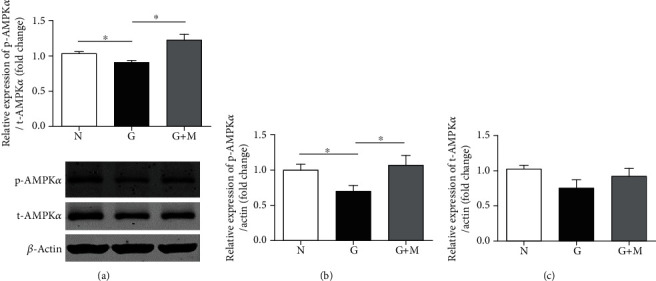
Metformin increased phospho-AMPK*α* in glyoxal-treated ARPE-19 cells. Glyoxal-induced ARPE-19 cells were cotreated with or without metformin (0.1 mM) for 5 min. The ratios of phospho-AMPK*α* to total-AMPK*α* (a), phospho-AMPK*α* to *β*-actin (b), and total-AMPK*α* to *β*-actin (c) were determined. Data are expressed as the mean ± SE (*n* = 7 in (a), *n* = 6 in (b), and *n* = 6 in (c), ∗*p* < 0.05 versus the glyoxal group). N: normal control; G: glyoxal-treated group; G+M: glyoxal and metformin cotreated group.

To explore the protective mechanisms of metformin, the AMPK/Sirtuin-1 (Sirt1) signaling pathway and the expression of nuclear factor erythroid-2-related factor 2 (Nrf2) and thioredoxin-interacting protein (TXNIP) were detected with Western blot. Compared with normal controls, the phosphorylation of AMPK*α* was significantly decreased, which had been increased by metformin (Figure 5). The phosphorylated AMPK*α* was decreased by 11.93% (*n* = 7, *p* < 0.05) and was increased by 35.43% (*n* = 7, *p* < 0.05) with metformin treatment. There were no significant differences of total-AMPK*α* among the three groups.

**Figure 6:**
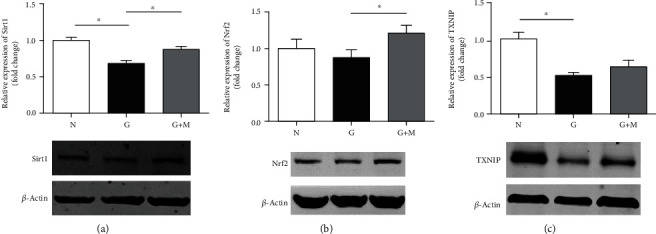
Metformin increased the expressions of Sirt1, Nrf2, and TXNIP in glyoxal-treated ARPE-19 cells. Glyoxal-induced ARPE-19 cells were cotreated with or without metformin (0.1 mM) for 24 h. Western blot and the quantification of Sirt1 (a), Nrf2 (b), and TXNIP (c). Data are expressed as the mean ± SE (*n* = 6 in (a), *n* = 8 in (b), and *n* = 7 in (c), ∗*p* < 0.05 versus glyoxal group). N: normal control; G: glyoxal-treated group; G+M: glyoxal and metformin cotreated group.

The protein expressions of Sirt1, Nrf2, and TXNIP were also decreased in glyoxal-treated ARPE-19 cells compared with that in normal controls, and metformin treatment was shown to increase these expressions (Figure 6). For example, compared with normal controls, the protein expressions of Sirt1, Nrf2, and TXNIP were decreased by 31.98% (*n* = 6, *p* < 0.05), 11.88% (*n* = 8, *p* > 0.05), and 47.87% (*n* = 7, *p* < 0.05), respectively, in the glyoxal-treated group, which were increased by 27.38% (*n* = 6, *p* < 0.05), 39.01% (*n* = 8, *p* < 0.05), and 22.09% (*n* = 7, *p* > 0.05), respectively, by metformin.

### 3.5. Metformin Upregulated Tight Junction Proteins in Glyoxal-Treated ARPE-19 Cells

**Figure 7:**
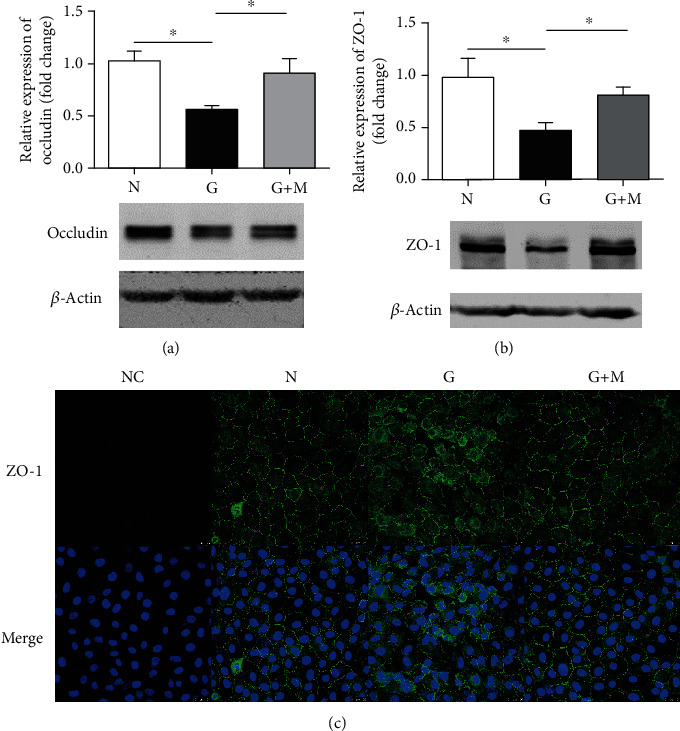
Metformin upregulated tight junction proteins in glyoxal-treated ARPE-19 cells. Glyoxal-induced ARPE-19 cells were cotreated with or without metformin (0.1 mM) for 24 h. Western blot and quantification of occludin (a) and ZO-1 (b). (c) Examination of ZO-1 immunostaining by confocal microscopy. Data are expressed as the mean ± SE (*n* = 6 in (a), *n* = 5 in (b), ∗*p* < 0.05 versus glyoxal group). NC: negative control; N: normal control; G: glyoxal-treated group; G+M: glyoxal and metformin cotreated group. Scale bar, 25 *μ*m.

To determine whether metformin treatment could maintain the barrier functions of RPE cells, we examined the tight junction proteins Occludin and ZO-1 with Western blot and immunofluorescence. The data showed that the protein expression of Occludin and ZO-1 were decreased in glyoxal-treated ARPE-19 cells and increased by metformin (Figure 7). Compared with normal controls, the protein expressions of Occludin and ZO-1 were decreased by 43.58% (*n* = 6, *p* < 0.05) and 52.73% (*n* = 5, *p* < 0.05), respectively, in glyoxal-treated ARPE-19 cells. Metformin treatment can increase their expression by 62.16% (*n* = 6, *p* < 0.05 for Occludin) and 71.94% (*n* = 5, *p* < 0.05 for ZO-1), respectively.

To further confirm metformin's effect, we performed fluorescence measurements of ZO-1 in ARPE-19 cells. As shown in Figure 7(c), the ZO-1 distribution was altered and disorganized in the glyoxal-treated group. However, metformin treatment could maintain the distribution pattern of tight junctions in the RPE.

### 3.6. Metformin Maintained Tight Junctions of the RPE in SI-Treated Rats

**Figure 8:**
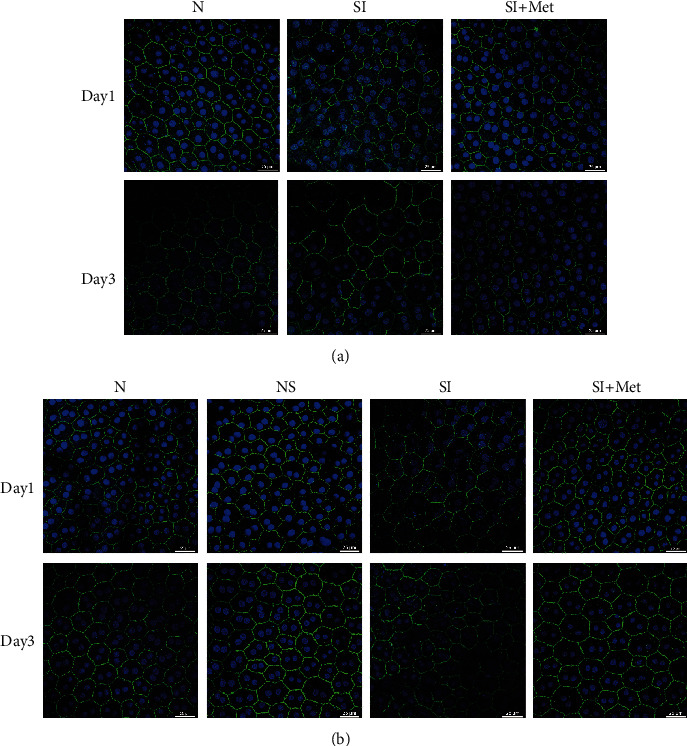
Metformin maintains the integrity of tight junctions of RPE cells in SI-treated rats. ZO-1 immunostaining (green) in RBCCs was examined at 1 day and 3 days after SI injection with or without intraperitoneal (a) or intravitreal (b) injections of metformin. N: intraperitoneal injections of normal saline (a) or vehicle (b); NS: intravitreal injections of normal saline; SI: sodium iodate-injection group; SI+Met: sodium iodate and metformin cotreatment group. Scale bar, 25 *μ*m.

To further confirm the maintenance of barrier functions of the RPE by metformin *in vitro* (Figure 7(c)), metformin was administered either intraperitoneally or intravitreally in SI-treated rats. As shown in Figure 8, compared with normal controls, the continuity of tight junctions, indicated by ZO-1 immunostaining, was disorganized/disrupted at 1 day and 3 days after SI injection. The morphology of RPE cells was largely changed in SI-treated rats; e.g., the cellular body was enlarged with more than two nuclei, and the number of RPE cells with characteristic hexagonal morphology was reduced, especially at 3 days. Metformin, delivered systemically (Figure 8(a)) or locally (Figure 8(b)), could maintain the integrity of barrier functions of the RPE and the RPE's characteristic morphology, indicating the RPE was protected from SI-induced oxidative stress by metformin.

## 4. Discussion

The irreversible vision loss caused by AMD greatly effects the quality of life of people worldwide. The clinical application of antivascular endothelial growth factor (VEGF) drugs helps preserve vision in wet AMD to a certain extent, but there are still no effective treatments for dry AMD. Therefore, the pathomechanisms of dry AMD have become a focus of research. In the present study, oxidative stress in RPE and the protection of metformin were explored in SI-treated rats and glyoxal-treated ARPE-19 cells. SI-induced rats have become a classic animal model that has been used to study the RPE and photoreceptor degeneration. Meanwhile, it has been reported that the reactive intermediate for the advanced glycation end products (AGEs) can form ROS, which ultimately leads to intracellular oxidative stress [19]. Compared with commonly used hydrogen peroxide (H_2_O_2_) and tert-butyl hydroperoxide (tBH, a stable form of H_2_O_2_), the glyoxal-treated model better mimics the age-related oxidative stress patterns associated with intracellular accumulation of AGEs in retinal diseases [19]. We therefore used SI to treat SD rats and glyoxal to treat ARPE-19 cells to induce oxidative stress, thus mimicking the pathogenesis of dry AMD, to study the effects of metformin.

The protective effects of metformin were extensively studied in many aging and senescence cellular models [20–22], as well as in hyperglycemia-treated ARPE-19 cell models [23]. In this study, we demonstrated that metformin prevented the SI-induced destruction of tight junctions *in vivo*. *In vitro*, metformin inhibited glyoxal-induced ARPE-19 cell death, reduced intracellular ROS production, and decreased the apoptosis rate. In addition to these findings, changes in intracellular NO levels are worth investigating. NO is an important gas signal molecule with diverse functions for maintaining retinal homeostasis. Under normal physiological conditions, NO synthesis and oxygen free radical degradation are in dynamic equilibrium [24]. When this balance is broken in a pathological state, excess free radicals can inactivate NO. As shown in clinical studies, lower NO levels were found in the plasma of AMD patients compared with the control group [24, 25]. The formation of peroxynitrite was considered as the reason for the decrease in NO caused by the increase in oxidative stress [26]. In this study, the level of NO was significantly increased in the metformin-treated group, suggesting metformin can increase the production of NO. A similar result was also shown in obese rats, confirming that metformin can enhance the release of NO. [27] Metformin itself may also be the source of NO, which provides exogenous supplements, because of its unstable N-N double bond structure. However, the exact role of NO in the onset of AMD is still undefined and needs further exploration. In some studies on AMD, iNOS-induced elevated NO levels have been found, as the enzyme can be stimulated by inflammation along with increased oxidative stress [28]. All of these results supported metformin playing a prominent role in preventing glyoxal-induced oxidative damage in ARPE-19 cells.

We further investigated the key factors that initiate the antioxidant activity of metformin. 5′-Adenosine monophosphate- (AMP-) activated protein kinase (AMPK) is an evolutionarily conserved serine/threonine protein kinase and serves as a cellular energy sensor. [29] The classical mechanisms of AMPK activation is a response to the decrease in cellular energy levels caused by various metabolic stresses or triggered by pharmacological activators, including metformin [30]. However, AMPK was inactivated in senescent cells; a H_2_O_2_-induced *in vitro* cell model demonstrated that oxidative stress-induced senescence resulted in a decrease in AMPK*α* [31]. In addition, AMPK activity was demonstrated to be lower in the RPE of patients with AMD, compared with the normal controls [32]. Similar results were also found in this study. When ARPE-19 cells were treated with glyoxal for 24 hours, the protein expression levels of p-AMPK*α* were downregulated. However, the downregulation was reversed by metformin. A number of studies extensively examined the neuroprotective effect of activation of AMPK signaling by metformin in neurodegenerative diseases, including Alzheimer's disease and Parkinson's disease [33]. These diseases are thought to share a common pathogenesis with AMD. The protective effect of other AMPK activators on oxidative damage of human RPE cells has also been demonstrated [34]. These data demonstrated that the antioxidant activity of metformin begins with the activation of AMPK.

SIRT1 is a nicotinamide adenosine dinucleotide- (NAD-) dependent histone deacetylase, which has the function of delaying aging and reducing age-related disorders [35]. Age-dependent oxidative stress can lead to DNA damage, which causes NAD^+^ depletion and decreased Sirt1 activity [36]. The authors' cell model also confirmed that the expression level of Sirt1 was significantly decreased in the glyoxal group. Sirt1 interacts with AMPK in regulating the oxidative metabolism. AMPK activates Sirt1 by enhancing the NAD^+^/NADH ratio and can be triggered by Sirt1 via promoting the deacetylation of liver kinase B1 (LKB1) [37]. As shown in our study, the level of p-AMPK*α* and Sirt1 shares a common trend in the glyoxal-treated group, and both can be reversed by metformin. The results supported the activation effect of metformin on the AMPK/Sirt1 pathway.

Nrf2 is considered to be the master regulator of the cellular antioxidant response, regulating the expression of antioxidant proteins by interacting with antioxidant response elements (ARE) [38]. It was regarded as a crucial downstream target of the AMPK/Sirt1 pathway to enhance resistance to oxidative stress [39, 40]. Many studies have shown that there is cross talk between the AMPK/Sirt1 pathway and Nrf2. The results indicated that metformin activates Nrf2 in an AMPK-dependent manner, and Nrf2 deficiency weakens the protection of metformin mediated by AMPK [41, 42]. However, it cannot be ignored that metformin can induce upregulation of Nrf2 when AMPK activity was blocked by compound C [43]. The exact relationship between them remains to be elucidated, but the metformin mediated upregulation of Nrf2, and the role in alleviating oxidative damage is certain. As shown in Figure 6(b), metformin significantly increased the expression level of Nrf2.

In general, TXNIP has been viewed as a redox switch that facilitates cellular oxidative stress by inhibiting thioredoxin and reducing oxidant-scavenging activity [44, 45]. However, this study and other studies found that, under oxidative stress, the expression level of TXNIP decreased sharply in RPE cells [46]. The mechanisms of TXNIP downregulation in RPE cells is thought to be related to autophagy and blood-retinal barrier (BRB) breakdown. Autophagy acts as a “housekeeper” to various stress responses and maintains cellular homeostasis [47]. Autophagic dysfunction in the RPE is thought to exacerbate oxidative stress and is associated with AMD susceptibility. [48] TXNIP depletion could suppress autophagic flux, but metformin was shown to be able to restore autophagy in ARPE-19 cells [49] and enhance autophagy via the AMPK/SIRT1 pathway to alleviate oxidative damage [50]. Our data indicates that the levels of p-AMPK*α* and TXNIP were simultaneously suppressed in the glyoxal-treated group, which suggested decreased autophagy activity in oxidative stress-induced ARPE-19 cells. The downregulation of TXNIP was further confirmed to negatively affect the BRB integrity by destroying RPE cell tight junctions in this study as well as others [46]. As shown in Figure 7, Occludin and ZO-1 were remarkably decreased in the glyoxal-treated group. In addition, the discreteness of cell boundaries and irregular shapes of RPE cells were obvious both *in vivo* and *in vitro*. Although the TXNIP levels were only slightly increased after treatment with metformin, the expression levels of p-AMPK*α* and tight junction proteins were remarkably increased. The results suggested that TXNIP may play a role as a switch, but not in a concentration-dependent manner. Still, the protective effect of metformin on the BRB is certain.

The current study has some limitations. For instance, glyoxal-treated ARPE-19 cells are not an ideal model to fully mimic all the facets of AMD. Therefore, it would be of great importance to establish an animal model for dry AMD and to validate the protective effect of metformin from more aspects. In addition, cross talk between the antioxidant pathway and autophagy pathway needs further exploration for a better understanding of the pathogenesis of AMD.

## 5. Conclusions

In conclusion, this study examined the protective effects of metformin in RPE cells against oxidative stress as well as maintenance of its barrier function in SI-treated rats and glyoxal-treated ARPE-19 cells. The results support the hypothesis that antioxidant and autophagy pathways are the cellular mechanisms elicited by metformin in the RPE. This information increases the understanding of how metformin may serve as a drug for the treatment of dry AMD.

## Data Availability

The authors follow the FAIR Guiding Principles for scientific data management and stewardship-findability, accessibility, interoperability, and reusability. The data used to support the findings of this study are included within the manuscript. However, additional information on this study may be obtained upon request to the corresponding author (13917311571@139.com, or fangliu_2004@yahoo.com).
